# Change of renal function after short-term use of cardioprotective agents in patients with type 2 diabetes is not accurately assessed by the change of estimated glomerular filtration rate: an observational study

**DOI:** 10.1186/s13098-022-00874-1

**Published:** 2022-07-21

**Authors:** Julie Kolwelter, Kristina Striepe, Agnes Bosch, Dennis Kannenkeril, Christian Ott, Mario Schiffer, Roland E. Schmieder

**Affiliations:** 1 Department of Nephrology and Hypertension, University Hospital Erlangen, Friedrich-Alexander University Erlangen-Nuremberg (FAU), Ulmenweg 18, 91054 Erlangen, Germany; 2 Department of Cardiology, University Hospital Erlangen, Friedrich-Alexander University Erlangen-Nuremberg (FAU), Erlangen, Germany; 3 Department of Nephrology and Hypertension, Paracelsus Medical School, Nuremberg, Germany

**Keywords:** Glomerular filtration rate, Inulin clearance, Type 2 diabetes, Change of renal function

## Abstract

**Background:**

After initiating cardioprotective agents, a fall of estimated glomerular filtration rate (eGFR) has been reported in several studies. Our goal was to evaluate the accuracy of change of Chronic Kidney Disease Epidemiology Collaboration (CKD-EPI) eGFR in patients with type 2 diabetes (T2D) after short-term pharmacological intervention with angiotensin-converting enzyme inhibitor, angiotensin-receptor blocker, gliptin or sodium-glucose cotransporter-2 inhibitor.

**Methods:**

We analyzed 190 patients with T2D in the early stage of the disease, having no overt renal impairment by CKD-EPI equation. In each patient, we measured GFR (mGFR) by applying the constant infusion input clearance technique with sinistrin (Inutest; Fresenius, Linz, Austria) at baseline and after short-term (4–12 weeks) pharmacological intervention with cardioprotective agents (ramipril, telmisartan, linagliptin, metformin, empagliflozin) that potentially lead to an alteration of renal function. Simultaneously, a standardized analysis of serum creatinine was performed and eGFR was estimated by the CKD-EPI equation.

**Results:**

Average mGFR was 111 ± 20 ml/min/1.73m^2^, whereas eGFR was lower with 93 ± 13 ml/min/1.73m^2^. The ratio eGFR/mGFR in relation to mGFR was almost curvilinear, showing an underestimation of renal function by eGFR in the upper normal range. At baseline only 80 patients (42%) lay within ± 10% of mGFR and the concordance correlation coefficient (CCC) was extremely low (− 0.07). After short-term pharmacological intervention changes in eGFR and mGFR correlated with each other (r = 0.286, p < 0.001). For example, for a given mGFR of 111 ml/min/1.73m^2^, a change of mGFR by ± 10% corresponded to ± 11 ml/min/1.73m^2^, but the confidence interval of eGFR was 25 ml/min/1.73m^2^. The CCC was low (0.22).

**Conclusion:**

The agreement between eGFR by CKD-EPI and mGFR is modest and the change of renal function after short-term pharmacological intervention is not accurately and precisely reflected by the change of eGFR in patients with T2D in the early stage of their disease.

## Introduction

Diabetes is, driven by its increasing prevalence, the leading cause for the development of chronic kidney disease (CKD) throughout the world, immediately followed by arterial hypertension [1]. Diabetic kidney disease (DKD) is a serious complication in patients with type 1 and type 2 diabetes (T2D) and is associated with higher cardiovascular and all-cause mortality than nondiabetic kidney disease [2]. In the early stage of renal involvement, glomerular hyperfiltration is often found in patients with type 1 (10–67%) and also type 2 diabetes (6–73%), preceding the onset of albuminuria, leading to a slow decline of renal function and finally to end-stage kidney disease [3, 4]. This disease course shows how important it is to detect early, in the stage without any apparent organ damage, hyper- and hypofiltration.

The current strategy to diagnose DKD is built on the presence of albuminuria and/or reduced glomerular filtration rate (GFR) assessed by estimating equations as well as the absence of any other causes of renal disorder (e.g. arterial hypertension or inherited kidney disease). However, in the recent decades, the prevalence of normoalbuminuric DKD, which features normal urine albumin to creatinine ratio (UACR) and reduced GFR, rises [5].

Clearance is calculated by estimating equations based on serum creatinine and serum cystatin C as well as age, ethnicity and gender. It was Paul Effersoe, who has introduced the first estimating equation in 1957, since then multiple equations for different populations have been developed [6]. The Modification of Diet in Renal Disease (MDRD) equation has been developed in people with CKD and as such, its limits lie in imprecision and underestimation in people with normal or high GFR levels [7]. In order to overcome this poor performance across a variety of populations, the MDRD equation has been replaced 10 years later by the Chronic Kidney Disease Epidemiology (CKD-EPI) equation [8], which has been updated most recently [9]. However, data suggest that the CKD-EPI estimating equation underperforms in patients with T2D and especially in patients with high GFR levels (glomerular hyperfiltration) [10, 11].

Measurement errors with the estimating equation occur when the estimating equation is applied in patients differing from the reference equation population [12]. For example, in a population of potential kidney donors with estimated GFR (eGFR) 45–59 ml/min/1.73m^2^, measured GFR (mGFR) is underestimated by 20% if the CKD-EPI equation is used, as the CKD-EPI equation was developed in a pooled cohort of mostly CKD patients with a small amount of healthier patients [13]. Serum cystatin C, which is not affected by muscle mass and diet, is an alternative endogenous marker to serum creatinine. Unfortunately in comparative studies, the cystatin C based equation was not more accurate than the creatinine based equation [14]. However, the combination of both markers in one equation delivers more accurate results in eGFR in diverse populations pooled from five prior conducted studies [15]. The 2021 updated CKD-EPI estimating equation combing serum creatinine and serum cystatin C showed minimal inaccuracy for both race groups (black persons and non-black persons) [9]. Unfortunately, serum cystatin C is not routinely measured in clinical practice due to its costs [16].

Estimating equations are not able to reflect the true renal function. They should however be able to detect, despite their lack of precision and accuracy, the change of renal function over time. The misinterpretation of alterations in eGFR might result in an early inappropriate discontinuation of the pharmacological therapy. Renal function evaluation should be part of each physician’s concern especially in patients with cardiovascular risk factors, heart failure or kidney disease. The best overall indicator of renal function is the GFR, which allows the improvement of initiation, adaption or discontinuation of the needed medical therapy and the stratification of the patients at risk of adverse outcome. The gold standard for measuring the GFR equals the average GFR measured by urine inulin clearance over a 24-h period, enabling to consider biorhythm fluctuations of GFR as well [17]. The simplified version measures the clearance of an exogenous marker such as inulin, iothalamate, iohexol or ^51^Cr-EDTA, which is exclusively and entirely eliminated by the glomeruli, however during a shorter period. The use of mGFR is limited to special applications due to its invasiveness, work intensity and cost and is therefore simply not feasible in clinical practice.

We focused exclusively on patients with T2D in the early stage of their disease, prior to macroalbuminuria or CKD stage 3 or higher, as it is important for clinicians and family doctors to detect early and reliably a change in renal function in daily clinical practice. Hence, the primary objective of this study was to evaluate the accuracy of the routinely used estimating equation CKD-EPI to detect short-term changes of renal function after pharmacological intervention with angiotensin-converting enzyme inhibitor, angiotensin-receptor blocker, gliptin or sodium-glucose cotransporter-2 inhibitor.

## Material and methods

### Study design

We conducted a retrospective, single-center, observational study in patients who participated in placebo-controlled, clinical trials (NCT00240422, NCT01835678, NCT02752113) between 2005 and 2018 in our clinical research center at the University Hospital Erlangen-Nuremberg. All clinical and laboratory parameters were acquired at baseline under the same conditions. According to the different study protocols, follow-up parameters were gathered after 4 weeks treatment with linagliptin (NCT 01835678), 9 weeks treatment with ramipril or telmisartan (NCT00240422) or 12 weeks treatment with the combination metformin/insulin or linagliptin/empaglifozin (NCT02752113), respectively.

### Study population

We analyzed a total of n = 190 patients with T2D, aged between 18 and 75 years, in the early stage of their disease defined as diabetes duration < 10 years, absence of macroalbuminuria and diabetic retinopathy, HbA1c < 10% and renal function estimated by the CKD-EPI equation ≥ 60 ml/min/1.73m^2^. Main exclusion criteria were uncontrolled arterial hypertension, any other form of diabetes than T2D and the use of insulin, glitazone, gliptin or SGLT2 inhibitor (prior to study participation). Due to the study protocols, all patients either were off antidiabetic medication or took their metformin medication for at least 3 months prior to the inclusion.

### Clinical parameters

Demographic data of all participants including medical history and concomitant medication were assessed at baseline in the same standardised manner in all patients.

Fasting venous blood samples were drawn to determine serum creatinine, fasting-plasma glucose and HbA1c. Serum creatinine level was measured using the modified Jaffe method with an isotope-dilution mass spectrometry (IDMS) correction factor and enabling thereby IDMS-traceable results. Fasting-plasma glucose was measured by the hexokinase method in a photometer and HbA1c was measured by turbidimetric inhibition immunoassay (TINIA).

In the early morning spot urine, the urinary albumin concentration was assessed by turbidimetric method and the urinary creatinine concentration was assessed photometrically by reaction of creatinine with picric acid (Jaffe method). UACR is the result of dividing the serum albumin concentration by the serum creatinine concentration. Microalbuminuria was defined as UACR between 30 and 300 mg/g creatinine.

### Estimated GFR (eGFR)

At each sinistrin clearance, GFR was estimated by using the most widely used estimating equation in clinical practice, the CKD-EPI equation. The CKD-EPI equation expressed as a single equation is [8]:

$$ \begin{aligned} {\text{eGFR }} = & { 141 } \times {\text{ min }}\left( {{\text{Scr }} \times \, 0.0{113}/{\text{k}},{1}} \right)^{{\text{a}}} \times {\text{ max }}\left( {{\text{Scr }} \times \, 0.0{113}/{\text{k}},{1}} \right)^{{ - {1}.{2}0{9}}} \times \, 0.{993}^{{{\text{age}}}} \\ & \times { 1}.0{18 }\left( {\text{if female}} \right) \, \times { 1}.{159 }\left( {\text{if black}} \right), \\ \end{aligned} $$

where k is 0.7 for women and 0.9 for men, α is − 0.329 for women and − 0.411 for men.

Scr is serum creatinine in mg/dl, min indicates the minimum of Scr/k or 1 and max indicates the maximum of Scr/k or 1.

We did not need to adjust for black race in our observational study, since all participants were Caucasians. Most recently the CKD-EPI estimation equation has been updated [9], but the publication became available after our analysis has been done. Nevertheless, we did sensitivity analysis by using the updated 2021 CKD-EPI estimating equation.

### Measured GFR–reference method (mGFR)

In each patient, the gold standard method, i.e. constant-infusion input steady state clearance technique with sinistrin (Inutest; Fresenius, Linz, Austria) without urine sampling was used for measuring GFR [18–20]. Under this steady state conditions, the amount of infused sinistrin is equal to the excreted amount of the tracer substance. By using the input steady state clearance technique, we avoided the need of bladder catherization and the dependency of a complete bladder emptying which is a serious cause of inaccuracy in some patients. All clearance examinations were performed with the patient in supine position, in a quiet and temperature-controlled laboratory and always at the same time in the morning (10 am). The method has been the same in all patients included in this analysis (NCT 01835678, NCT00240422, NCT02752113).

In brief, we placed an intravenous line in each arm of the patient, one for the infusion and, on the opposite arm, one for drawing blood samples. After a bolus infusion of sinistrin, an inulin-type β-d-fructan, over 15 min, a constant infusion was given subsequently over 105 min to achieve the above mentioned steady state conditions. Blood samples to determine the concentration of the tracers and consequently mGFR were drawn at 0, 115 and 120 min in duplicate (coefficient variation < 5%) [21]. Sinistrin was measured indirectly by converting the inulin-type β-d-fructan to fructose and subsequently measuring fructose by an enzymatic method (Boehringer Mannheim, Mannheim, Germany).

For further analyses, in order to be able to compare the results of mGFR and eGFR, the clearance results were indexed to 1.73m^2^ body surface area (BSA) according to the Mosteller formula: [22] BSA = (height × weight)^1/2^ /3600.

### Definition of glomerular hyperfiltration

Despite vast interest in glomerular hyperfiltration, reported to be a predictor of DKD, a precise definition in the literature is missing. According to a systematic review of glomerular hyperfiltration assessment published in 2015, comprising 405 studies, glomerular hyperfiltration is defined as mGFR by inulin clearance (cut-off value according to the evaluation of 38 studies) above 138 ± 10 ml/min/1.73m^2^ and as eGFR by estimating equations (cut-off value according to the evaluation of 26 studies) above 128 ± 15 ml/min/1.73m^2^ [23]. The mentioned cut-off values are used in our observational study in order to identify the patients with glomerular hyperfiltration.

### Statistical analysis

Depending on data distribution, data were expressed as mean ± standard deviation (SD) or median and interquartile range (IQR). The Bland–Altman plot was used as descriptive tool to evaluate the agreement between two methods, showing the relationship between the difference of mGFR and eGFR and the mean of the two methods. The limit of agreement comprises the differences between two measurements lying in the reference interval defined as mean difference ± 1.96 × SD. If the two methods are comparable, the differences should be small and close to 0 [24]. Precision and accuracy of both methods were evaluated by Lin’s concordance correlation coefficient (CCC) [25]. CCC equals the product of the Pearson correlation coefficient and a bias correction factor. The Pearson correlation coefficient measures how far each observation deviates from the best-fit line and is a measure of precision, whereas the bias correction factor measures how far the best-fit line deviates from the 45° line through the origin and is a measure of accuracy. A CCC > 0.9 reflects optimal concordance between the two measurements, a CCC of 0 reflects no concordance at all. Further, relative accuracy was described as the percentage of eGFRs falling within ± 10% (P10), ± 20% (P20) and ± 30% (P30) of a given mGFR value, respectively. All analyses were performed using SPSS statistics 28 (IBM Corporation, Chicago, IL, USA).

## Results

A total of 190 patients with T2D, aged 59 ± 9 years (min–max, 29–75 years) of whom 135 (71%) were men, were included. Of note, at the time of renal clearance measurements, median value for known diabetes duration was 5.4 years (IQR 2.6–8.6 years) and mean value for HbA1c 7.1 ± 0.9%. They all had well-controlled arterial hypertension and only 21 (11%) patients had microalbuminuria. Fifty-six (29%) patients received an angiotensin-converter enzyme inhibitor (18%) or an angiotensin receptor blocker (11%). Further clinical characteristics of the study participants are displayed in Table 1.

**Table 1:** Baseline characteristics of all study participants

	All participants (n = 190)
Age (years)	59 ± 9
Sex (n, %)	Male: 135 (71%) Female: 55 (29%)
BMI (kg/m^2^)	30.1 ± 4.6
Body surface area (m^2^)	2.1 ± 0.2
Systolic blood pressure (mmHg)	137 ± 14
Diastolic blood pressure (mmHg)	81 ± 9
FP-glucose (mg/dl)	158 ± 46
HbA1c (%)	7.1 ± 0.9
Serum creatinine (mg/dl)	0.81 ± 0.15
mGFR indexed to BSA (ml/min/1.73m^2^)	111 ± 20
eGFR by CKD-EPI (ml/min/1.73m^2^)	93 ± 13
UACR (mg/g creatinine)	10.3 (6.8–21.2)
Microalbuminuria (n, %)	21 (11%)
Diabetes duration (years)	5.4 (2.6–8.6)

Data are mean ± SD or median (interquartile range, IQR). BMI, body mass index; FP-glucose, fasting-plasma glucose; HbA1c, glycated haemoglobin; mGFR, measured glomerular filtration rate; BSA, body surface area; eGFR, estimated glomerular filtration rate; CKD-EPI, Chronic Kidney Disease Epidemiology Collaboration; UACR, urine albumin to creatinine ratio

### Agreement between mGFR and eGFR at baseline

**Fig. 1:**
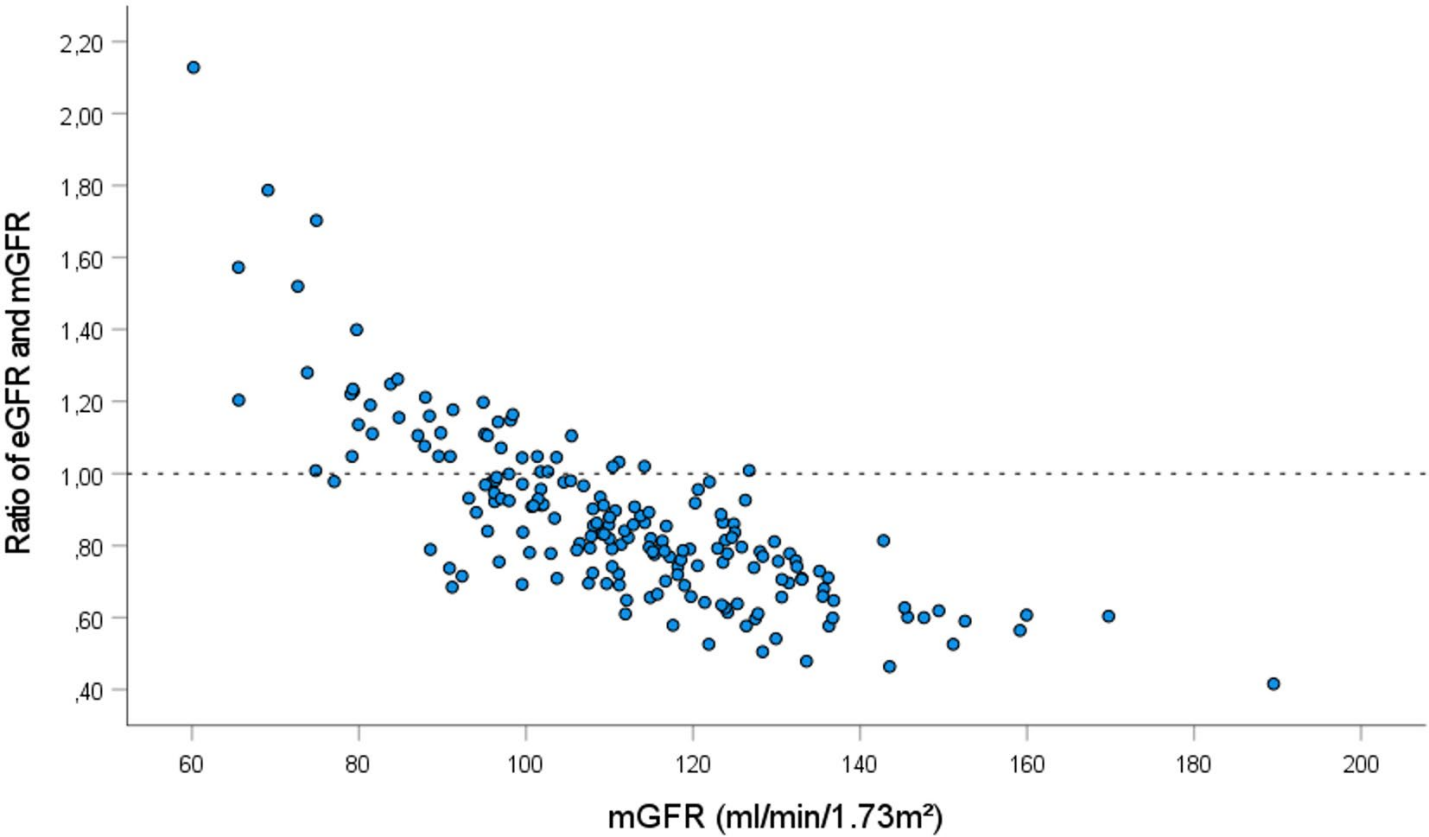
Ratio of eGFR and mGFR in relation to mGFR (ml/min/1.73m^2^). The dotted line represents a ratio of 1, indicating that eGFR corresponds to mGFR

At baseline, average mGFR was 111 ± 20 ml/min/1.73m^2^ and was higher than average eGFR by CKD-EPI with 93 ± 13 ml/min/1.73m^2^ (p < 0.001). We calculated the ratio of eGFR and mGFR in relation to mGFR. Ideally, the ratio equals 1, indicating that eGFR corresponds to mGFR, i.e. reflecting accurately renal function. As illustrated in Fig. 1, the ratio of eGFR and mGFR in relation to mGFR was almost curvilinear explaining the underestimation of mGFR in the upper normal range of renal function by eGFR, as indicated by most points lying below 1.

The parameter P30 describes the percentage of eGFR that lies within ± 30% of a given mGFR value. Taking the mean value of mGFR, namely 111 ml/min/1.73m^2^, any eGFR between 78 and 144 ml/min/1.73m^2^ is within the ± 30% limit, which comprises n = 171 patients (90%) of the study cohort. However, if we take the parameter P10 as guidance (which is often done in clinical practice), any eGFR between 100 and 122 ml/min/1.73m^2^ is within the ± 10% limit, which comprises only 80 patients (42%) of the study cohort. The results of the additionally calculated P15, P20 and P25 values are shown in Table 2.

**Table 2:** Relative accuracy of estimated glomerular filtration rate (eGFR)

Relative accuracy	n (%)	For a given mGFR of 111 ml/min/1.73m^2^, the range of mGFR (ml/min/1.73m^2^) equals
P10**	80 (42%)	100 to 122
P15**	121 (64%)	94 to 128
P20**	145 (76%)	89 to 133
P25**	161 (85%)	83 to 139
P30*	171 (90%)	78 to 144

^*^P30 is described as the percentage of estimated glomerular filtration rates (eGFR) that lie within ± 30% of the measured glomerular filtration rate (mGFR)

^**^P10, P15, P20 and P25 corresponds to a limit of error of ± 10%, ± 15%, ± 20% and ± 25%

**Fig. 2:**
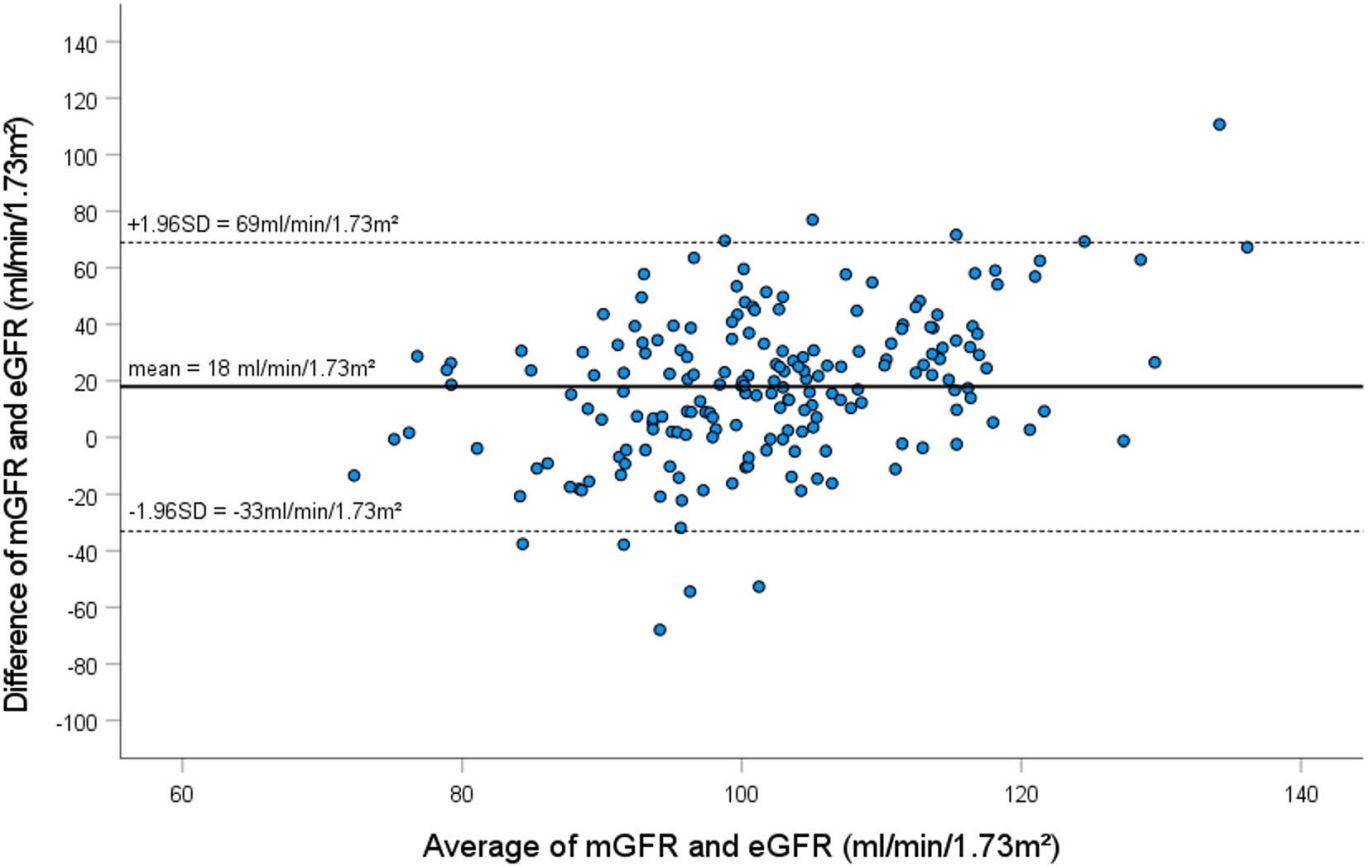
Bland Altman plot of eGFR by CKD-EPI versus mGFR by sinistrin clearance: difference between mGFR and eGFR (on y axis) versus mean of mGFR and eGFR (on x axis). The solid line and the dashed line indicate mean difference and 95% limits of agreement, respectively

Statistically, the accuracy of two methods can be assessed by the Lin’s CCC. A CCC of − 0.07 was calculated for the CKD-EPI equation at baseline, thereby indicating that there is no agreement. As illustrated in Fig. 2, the Bland Altman plot shows wide limits of agreement ranging from − 32 to 69 ml/min/1.73m^2^. Many points lie far from the mean line, which indicates again poor agreement between mGFR and eGFR at baseline.

### Glomerular hyperfiltration

At baseline, 12 (6.3%) of 190 patients presented glomerular hyperfiltration according to mGFR, whereas only one patient (0.5%) showed glomerular hyperfiltration according to eGFR. After the short-term pharmacological intervention, we identified glomerular hyperfiltration according to mGFR in 15 (7.9%) patients and according to eGFR in only one patient (0.5%).

### Agreement between changes of mGFR and eGFR

**Fig. 3:**
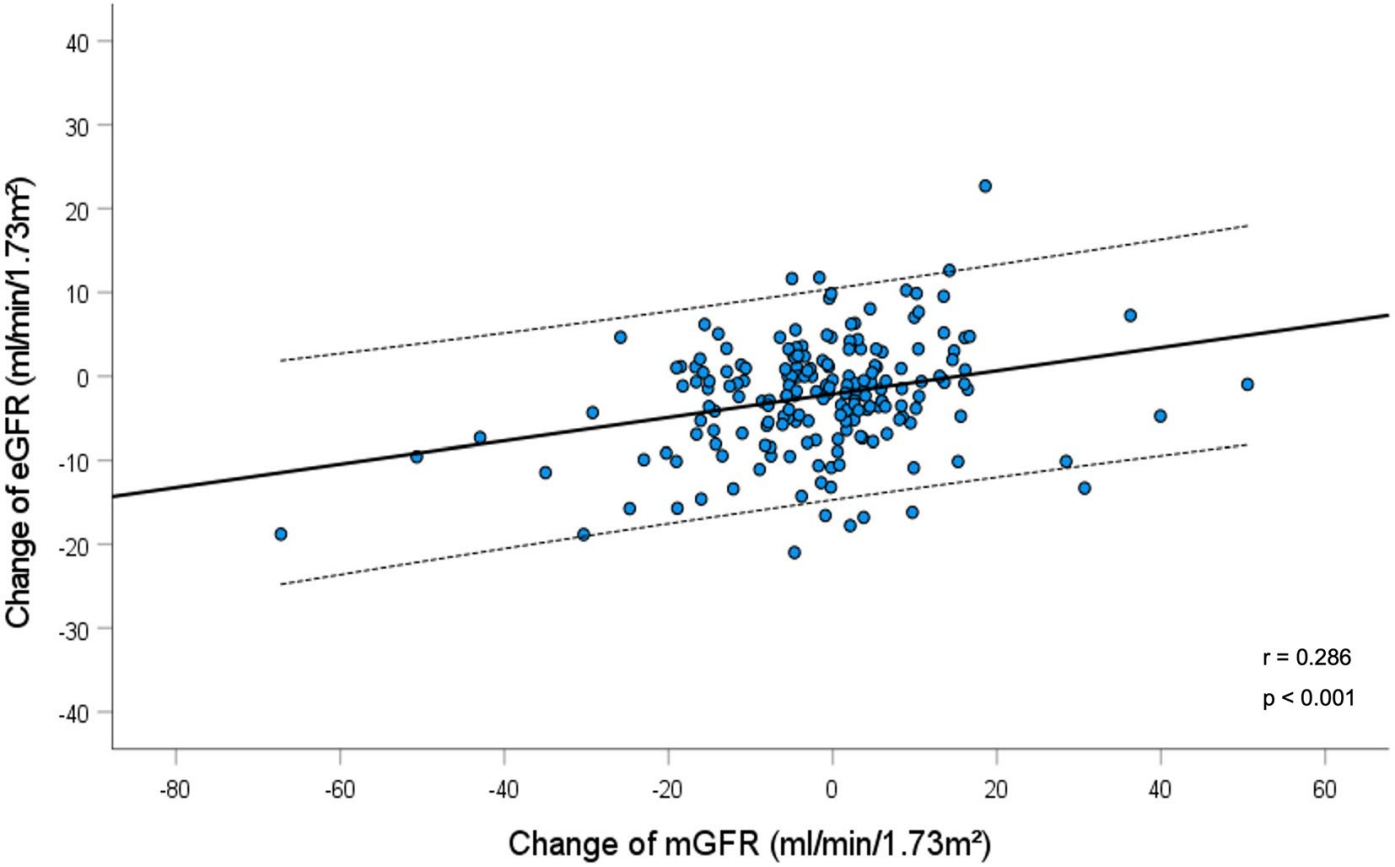
Change of eGFR (ml/min/1.73m^2^) in relation to change of mGFR (ml/min/1.73m^2^) after short-term pharmacological intervention. The solid line represents the regression line. The dashed lines represent 95th confidence interval

**Fig. 4:**
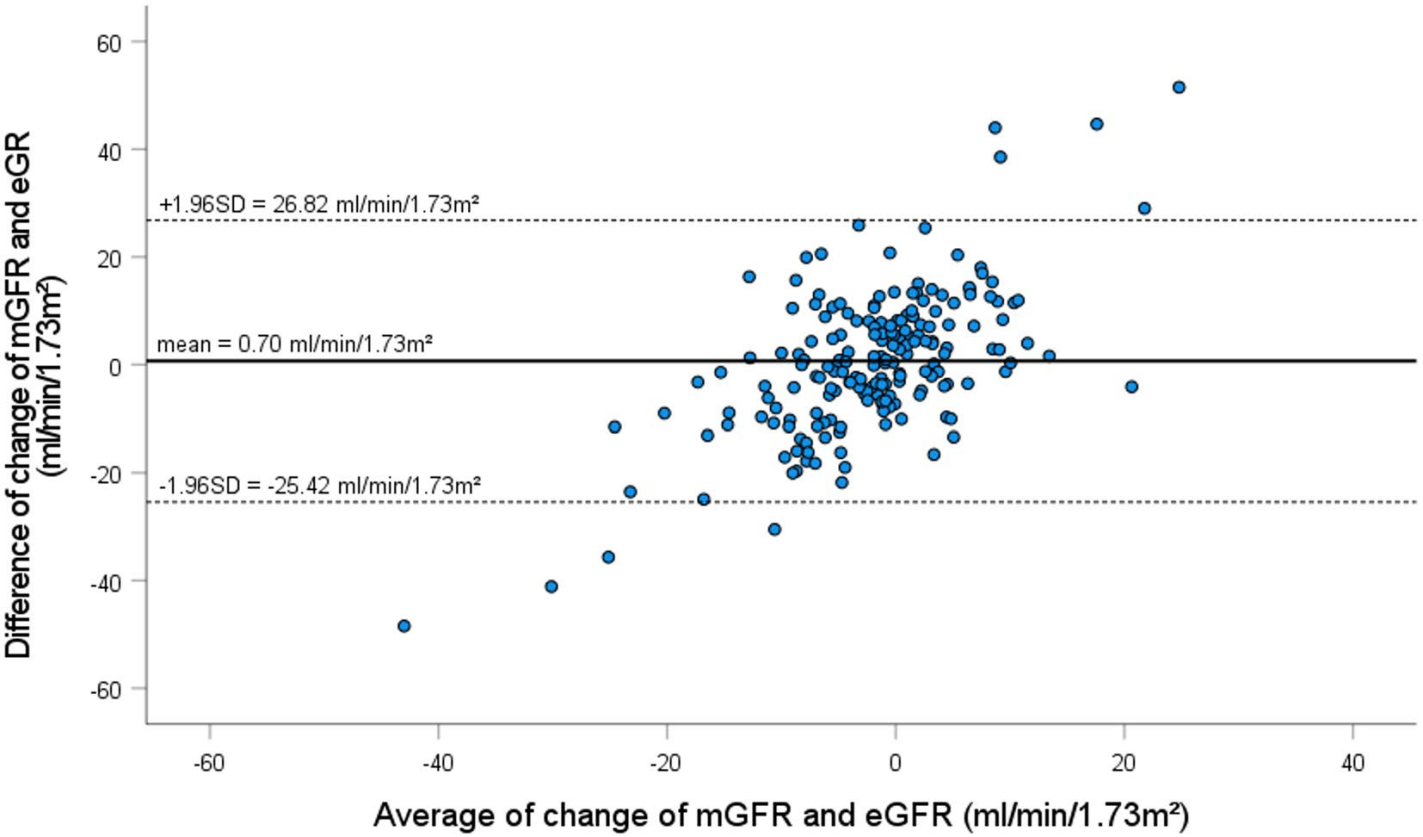
Bland Altman plot of change of eGFR by CKD-EPI versus change of mGFR by sinistrin clearance: difference between change of mGFR and change of eGFR (on y axis) versus average of change of mGFR and change of eGFR (on x axis). The solid line and the dashed line indicate mean difference and 95% limits of agreement, respectively

All patients underwent a short-term pharmacological intervention with angiotensin-converting enzyme inhibitor, angiotensin-receptor blocker, gliptin and sodium-glucose cotransporter-2 inhibitor, susceptible to lead to potential changes of renal function. The average mGFR change was − 1.65 ± 14 ml/min/1.73m^2^ and the average eGFR change was slightly greater with − 2.35 ± 6.6 ml/min/1.73m^2^ (p = 0.470). Changes in eGFR and mGFR correlated with each other (r = 0.286, p < 0.001), but with wide 95% confidence intervals (Fig. 3). For a given value of mGFR of 111 ml/min/1.73m^2^, a change of mGFR by ± 10% corresponds to ± 11 ml/min/1.73m^2^ on average, but the confidence interval of eGFR with 25 ml/min/1.73m^2^ was wide. To give an example, a mGFR change of − 11 ml/min/1.73m^2^ corresponds to an eGFR change of − 3.5 ml/min/1.73m^2^ with a 95% CI from − 16.3 to + 9 ml/min/1.73m^2^ and a mGFR change of + 11 ml/min/1.73m^2^ corresponds to an eGFR change of − 0.5 ml/min/1.73m^2^ with a 95% CI from − 13 to + 12 ml/min/1.73m^2^. In accordance, and as illustrated in Fig. 4, the Bland Altman plot shows wide limits of agreement ranging from − 25 to + 27 ml/min/1.73m^2^, with many points lying far away from the mean line, which indicates poor agreement between mGFR and eGFR change. Further, the calculated CCC for the eGFR change was 0.22, thereby indicating no agreement between the two measurement methods for the assessment of changes in renal function supposed to track short-term changes of renal function.

Of the 190 patients, 105 (55.3%) patients showed a lowered mGFR after short-term pharmacological intervention with ramipril, telmisartan, linagliptin, metformin or empagliflozin. In this subgroup, average baseline mGFR was 113 ± 18 ml/min/1.73m^2^ and average mGFR at the follow-up was 103 ± 16 ml/min/1.73m^2^ (average change of − 10 ± 11 ml/min/1.73m^2^, p < 0.001). The eGFR average values were different with baseline eGFR of 94 ± 11 ml/min/1.73m^2^ and follow-up eGFR of 91 ± 14 ml/min/1.73m^2^ (average change of − 3 ± 7 ml/min/1.73m^2^, p < 0.001). In this subgroup, the change of mGFR was significantly different from the change of eGFR (p < 0.001).

We did a sensitivity analysis by using the updated 2021 CKD-EPI estimating equation which showed no directional change of our results.

## Discussion

The aim of our study was to investigate the accuracy of assessing changes in renal function after short-term pharmacological intervention with cardioprotective agents by using eGFR and mGFR in patients with T2D. The study population is very special as the patients were in the early stage of the disease without overt nephropathy. Clinicians use estimating equations to track changes in eGFR after initiating several pharmacological agents susceptible to alter renal function. The concordance between short-term alteration in eGFR and alteration in mGFR as the gold standard measurement of renal function was not analyzed by others before. Studies compared iothalamate clearance with different estimating equations based on serum creatinine measurement over time and found that estimating equations approximate the change in iothalamate measured GFR in patients with type 1 diabetes [26]. However, we found in our patients with T2D, after short-term pharmacological intervention, no reliable detection of eGFR change by the CKD-EPI equation, although the average changes in mGFR and eGFR were close (− 1.65 ± 13.61 ml/min/1.73m^2^ and − 2.35 ± 6.62 ml/min/1.73m^2^, respectively). However, if we consider an given change of mGFR by ± 10% from baseline after initiating a new pharmacological intervention, a wide range of eGFR changes was observed, indicating no acceptable agreement between the two measurement methods. The CCC was low with 0.22, similar to the CCC of 0.28, found by others, by comparing long-term eGFR by CKD-EPI decline over years (median 4 years) to iohexol plasma clearance decline in patients with T2D [10]. We focused, in contrary to those long-term analyses, on short-term changes in eGFR, often observed after newly administrated pharmacological therapy, and often leading to withdrawal of the new initiated medical therapy. We observed that the CKD-EPI equation is less accurate in uncovering changes of renal function after short-term pharmacological intervention than mGFR. One possible explanation, even though the short interval argues against it, are potential biases from determinants of serum creatinine, e.g. such as change in body composition. Our data suggest that one single measurement of eGFR after an intervention that potentially affects renal function is not accurate and reliable. It remains to be determined whether additional measurements allow a more precise judgement of renal function.

Furthermore, we found that eGFR did not detect patients with T2D and presenting glomerular hyperfiltration. These patients constitute however an important group of patients with high therapeutic potential. Despite lots of research and broad interest in glomerular hyperfiltration, a stringent definition is absent in literature. We used the definite values for mGFR (> 138 ± 10 ml/min/1.73m^2^) and eGFR (> 128 ± 15 ml/min/1.73m^2^) published 2015 in a systematic review of glomerular hyperfiltration assessment. At baseline and at the follow-up, only one patient was detected having glomerular hyperfiltration according to eGFR, but mGFR was far from the glomerular hyperfiltration threshold (> 138 ml/min/1.73m^2^) with 60.2 ml/min/1.73m^2^ and 79.6 ml/min/1.73m^2^, respectively. Glomerular hyperfiltration occurs in the early phase of DKD in both type 1 and type 2 diabetes and constitutes an independent risk factor for advanced GFR reduction. Especially early detection of these patients is crucial [27]. Identical results with estimating equations failing to identify patients with glomerular hyperfiltration were found elsewhere [10].

Another result of our analysis was that the agreement between mGFR and eGFR was modest. Our study is the first to compare mGFR by sinistrin (Inutest) clearance with eGFR by CKD-EPI in patients with T2D, in opposite to others who have compared iohexol, ^51^Cr-EDTA and ^99m^Tc-DPTA with other estimating equations [28]. Factors influencing the creatinine-based equations are the variable synthesis of creatinine, affected by dietary intake and muscle mass, and secretion and reabsorption of creatinine by renal tubular cells. Overall, the CKD-EPI equation underestimates the true GFR in patients with T2D and eGFR ≥ 60 ml/min/1.73m^2^, especially in the upper normal range. This finding is in accordance with other previous published studies [29, 30]. The reason for this underestimation of GFR in patients with T2D and no overt nephropathy is still unclear. Patients’ characteristics such as age, sex, BMI, HbA1c or fasting plasma glucose levels provide no clear explanation [31].

Accuracy, reflected by all eGFR values within ± 30% (P30), was 90% for the CKD-EPI equation in our study population, indicating that only 10% of the eGFR values differ by more than 30% of mGFR. In order to be considered as an adequate tool for medical decisions, the accuracy level of P30 should outreach 75% and optimally 90% [32, 33]. In our cohort, the CKD-EPI equation reflects accurately mGFR and is in synchrony with the original work of the CKD-EPI study group where P30 was 80.4%. However, the 30% deviation is extremely wide and enables, in our cohort, an eGFR range from 78 to 144 ml/min/1.73m^2^. In patients with T2D, several studies have evaluated the agreement between mGFR and eGFR with variable results with P30 ranging from 81 to 90 for the CKD-EPI equation [15, 31, 34, 35]. Since P30 values vary widely, a move to a narrower limit of error (P20, P10) seems premature. Notably, the proportion of eGFRs within ± 10% of mGFR does not exceed 42%. Additionally, CCC for the CKD-EPI equation was extremely low, demonstrating that accuracy and precision of this estimating equation is poor in our study cohort.

One strength of the present study is the use of sinistrin (Inutest) clearance as reference method and known as gold standard, the standardized protocol in its execution and the fact that we included a homogenous study population.

Our study had several limitations. The population comprised only Caucasians in the very early stage of T2D without retinopathy and with well-controlled arterial hypertension and thereby allows no extension to other ethnicities, patients with type 1 diabetes as well as those with uncontrolled arterial hypertension. We did not apply the 2021 CKD-EPI equation as our analysis was performed before the updated 2021 version of the CKD-EPI equation was published [9]. However, we did a sensitivity analysis with the 2021 CKD-EPI equation and found no directional change of the results compared to the results obtained using the 2009 CKD-EPI equation. We also did not incorporate the assessment of cystatin C in our analysis, since cystatin C is not routinely measured due to its costs [16].

## Conclusion

In our cohort of patients with T2D and normal renal function, changes in eGFR by CKD-EPI after short-term pharmacological intervention with cardioprotective agents failed to accurately and reliably reflect changes in renal function measured by mGFR. In addition, eGFR by CKD-EPI did not detect glomerular hyperfiltration. The accuracy of eGFR equations needs improvement. Whether repeated measurements of eGFR in clinical practice improves accuracy needs to be determined.

## Data Availability

The datasets that support the findings of this study are available from the corresponding author upon request.

## Abbreviations

^51^Cr-EDTA: Chromium-51 labeled ethylenediamine tetraacetic acid
BSA: Body surface area
CCC: Concordance correlation coefficient
CI: Confidence interval
CKD: Chronic kidney disease
CKD-EPI: Chronic Kidney Disease Epidemiology
DKD: Diabetic kidney disease
eGFR: Estimated glomerular filtration rate
GFR: Glomerular filtration rate
HbA1c: Glycated haemoglobin
IDMS: Isotope dilution mass spectrometry
IQR: Interquartile range
MDRD: Modification of diet in renal disease
mGFR: Measured glomerular filtration rate
SD: Standard deviation
SGLT2 inhibitor: Sodium Glucose Co-Transporter-2 inhibitor
T2D: Type 2 diabetes mellitus
^99m^Tc-DPTA: Technetium-99 m labeled diethylenetriamine pentaacetic acid
UACR: Urine albumin to creatinine ratio
